# The Food Additive Benzaldehyde Confers a Broad Antibiotic Tolerance by Modulating Bacterial Metabolism and Inhibiting the Formation of Bacterial Flagella

**DOI:** 10.3390/ijms25168843

**Published:** 2024-08-14

**Authors:** Xia Xiao, Can Ma, Han Zhang, Wei Liu, Yanhu Huang, Chuang Meng, Zhiqiang Wang

**Affiliations:** 1 College of Veterinary Medicine, Yangzhou University, Yangzhou 225009, China; xiaoxia@yzu.edu.cn (X.X.); macan18652592290@outlook.com (C.M.); m15609938315@163.com (H.Z.); lw15264770396@163.com (W.L.); dx120230185@stu.yzu.edu.cn (Y.H.); 2 Jiangsu Co-Innovation Center for Prevention and Control of Important Animal Infectious Diseases and Zoonoses, Yangzhou 225009, China; 006503@yzu.edu.cn; 3 Jiangsu Key Laboratory of Zoonosis, Yangzhou University, Yangzhou 225009, China; 4 Institute of Comparative Medicine, Yangzhou University, Yangzhou 225009, China

**Keywords:** antibiotic, tolerance, benzaldehyde, *Escherichia coli*, ciprofloxacin

## Abstract

The rise of antibiotic tolerance in bacteria harboring genetic elements conferring resistance to antibiotics poses an increasing threat to public health. However, the primary factors responsible for the emergence of antibiotic tolerance and the fundamental molecular mechanisms involved remain poorly comprehended. Here, we demonstrate that the commonly utilized food additive Benzaldehyde (BZH) possesses the capacity to induce a significant level of fluoroquinolone tolerance in vitro among resistant *Escherichia coli*. Our findings from animal models reveal that the pre-administration of BZH results in an ineffective eradication of bacteria through ciprofloxacin treatment, leading to similar survival rates and bacterial loads as observed in the control group. These results strongly indicate that BZH elicits in vivo tolerance. Mechanistic investigations reveal several key factors: BZH inhibits the formation of bacterial flagella and releases proton motive force (PMF), which aids in expelling antibiotics from within cells to reducing their accumulation inside. In addition, BZH suppresses bacterial respiration and inhibits the production of reactive oxygen species (ROS). Moreover, exogenous pyruvate successfully reverses BZH-induced tolerance and restores the effectiveness of antibiotics, highlighting how crucial the pyruvate cycle is in combating antibiotic tolerance. The present findings elucidate the underlying mechanisms of BZH-induced tolerance and highlight potential hazards associated with the utilization of BZH.

## 1. Introduction

The utilization of antibiotics has been pivotal in the progression of contemporary medicine. However, due to the excessive and inappropriate use of antibiotics in medical settings and agriculture, bacteria have developed various mechanisms to combat their effects [1]. One particularly concerning mechanism is the emergence of antibiotic resistance through mutant or resistant genes. Identifying resistance becomes relatively straightforward as the minimum inhibitory concentration (MIC) of antibiotics increases in resistant bacteria. Another strategy bacteria employ is antibiotic tolerance, which enables them to survive for an extended period after exposure to antibiotics without any change in MICs [1,2]. Antibiotic tolerance often goes unnoticed due to the absence of standardized quantitative indicators, yet it significantly contributes to chronic and recurrent infections [3]. Furthermore, studies have demonstrated that antibiotic tolerance accelerates the development and evolution of drug resistance, emphasizing the need for increased attention toward understanding its formation [4]. Therefore, it is crucial to understand the factors contributing to antibiotic tolerance and identify the underlying molecular mechanisms.

An increasing number of studies have demonstrated a strong correlation between the physiological and metabolic changes in bacteria and their ability to tolerate antibiotics [5]. Bacteria in a state of low metabolic activity during the stationary phase exhibit higher tolerance towards bactericidal antibiotics [6]. Conversely, enhancing the basal respiration rate of *E. coli* through genetic modifications enhances the efficacy of bactericidal antibiotics compared to wild-type cells [7]. Additionally, exposure to specific environmental conditions such as poor nutrition can also result in the development of tolerance [8]. Studies have shown that genetic mutations can lead to the acquisition of antibiotic tolerance [9]. For instance, when the sodium–proton antiporter gene *nhaA* is deleted, *E. coli* exhibits inheritable tolerance [10]. Mutations in genes associated with growth defects including aminoacyl-tRNA synthetase (*metG*), ribose bisphosphate kinase (*prs*), ATP-dependent proteases (*clpX*, *clpP*), and a putative toxin–antitoxin module (*vapBC*) also lead to tolerance [9,11,12]. Additionally, multidrug tolerance in *E. coli* is mediated by both the toxin–antitoxin (TA) system *hipAB* [13,14] and two-component system *ZraPSR* [15]. Zhihui Lyu discovered that antibiotics exhibit higher tolerance in *Salmonella* cells lacking flagella [16]. Similarly, Jules et al. reported that *Pseudomonas aeruginosa* with deficiencies in flagella proteins such as *FlgE* also exhibited antibiotic tolerance [17], indicating an important role for flagella in this context. Alarmingly, external environmental factors or compounds can induce antibiotic tolerance as well. It has also been documented that potassium sorbate, a commonly used food additive, induces significant tolerance to fluoroquinolones in bacteria harboring the mobile colistin resistance gene *mcr* by inhibiting aerobic respiration and impeding antibiotic uptake [18]. Other studies have shown that sodium dehydroacetate, a food additive, can induce bacterial tricarboxylic acid cycle reconstruction, thereby inducing antibiotic tolerance [19]. Another food additive citric acid enhances broad-spectrum antibiotic tolerance by modulating bacterial metabolism and oxidative stress levels [20]. These findings underscore potential risks associated with food additives concerning antibiotic resistance.

Benzaldehyde (BZH), a derivative of benzoic acid, can be naturally found and chemically synthesized. The FAO/WHO (CAC/MISC 6–2013) has officially recognized its application as a flavor enhancer in the food industry, as well as in cosmetics and pharmaceutical products. It is also approved as a denaturing agent, solvent, and scent component in perfumery [21]. For substances such as benzoic acid, and its derivatives in the form of salts and parabens, including BZH, the ADI established by FAO/WHO (1996) is 5 mg/kg body weight/day or lower (expressed as benzoic acid equivalents) [22]. The dose was subsequently verified [23] and is still in place today [23]. But even with BZH’s widespread usage in the food and pharmaceutical industries, its effect on antibiotic therapy’s effectiveness remains unclear.

This study aims to explore the potential impact of BZH on the emergence of antibiotic tolerance and uncover the underlying molecular mechanisms. Surprisingly, our results suggest that the presence of BZH leads to the development of tolerance towards fluoroquinolone antibiotics in bacteria that are already resistant. This phenomenon was observed not only in controlled laboratory conditions but also within living organisms. The discovery emphasizes a novel detrimental dietary source, BZH, in relation to antibiotic tolerance. In terms of mechanism, BZH hinders aerobic respiration by consuming pyruvic acid and disrupts flagella function as well as antibiotics uptake by dissipating PMF. Additionally, it has been observed that BZH inhibits the generation of reactive oxygen species (ROS) induced using bactericidal antibiotics through the enhancement of superoxide dismutase (SOD) activity.

## 2. Results

### 2.1. Benzaldehyde Impairs Antibiotic Activity against Resistant Bacteria

**Figure 1:**
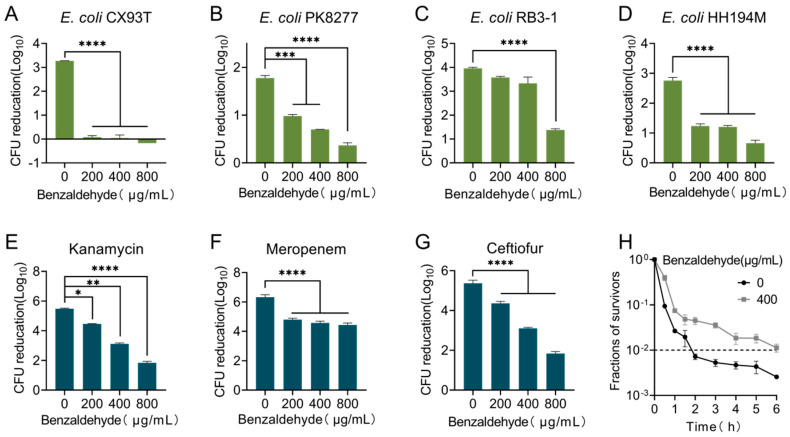
The bactericidal activity of antibiotics against pathogens is reduced by the presence of benzaldehyde. (**A**–**D**) Pre-culturing benzaldehyde with bacteria for 3 h resulted in a dose-dependent decrease in the effectiveness of ciprofloxacin against wild-type strains that are resistant to ciprofloxacin. (**E**–**G**) Benzaldehyde reduced the bactericidal activity of three bactericidal antibiotics (kanamycin, meropenem, and ceftiofur) against *E. coli* CX93T. (**H**) The minimum duration to achieve a 99% reduction in the bacterial population (MDK_99_) of ciprofloxacin against *E. coli* CX93T. All data obtained from biological experiments conducted in triplicate are reported as mean values with standard deviations (±SD). Statistical significance was determined using non-parametric one-way ANOVA, and the levels of significance were denoted as * *p* < 0.05, ** *p* < 0.01, *** *p* < 0.001, and **** *p* < 0.0001.

To investigate the impact of benzaldehyde (BZH) on bacterial susceptibility to antibiotics, we initially determined the minimum inhibitory concentration (MIC) of BZH against various strains. The results indicated that BZH did not exhibit direct inhibitory activity against Gram-negative bacteria, with an MIC greater than 1024 μg/mL (Table S1). Subsequently, we examined the influence of co-culturing bacteria with gradually escalating levels of BZH, spanning from 0 to 800 μg/mL, on bacterial growth. Surprisingly, no discernible effect on bacterial growth was observed (Figure S1). The MIC of ciprofloxacin against *E. coli* CX93T and *E. coli* RB3-1 was determined to be 16 μg/mL, while the MIC against *E. coli* PK8277 and *E. coli* HH194M was found to be 32 μg/mL. We then evaluated the bactericidal effectiveness of ciprofloxacin through quantifying the reduction in colony-forming units (CFUs) after co-incubation with BZH. Intriguingly, our findings revealed a dose-dependent attenuation in the bactericidal activity of ciprofloxacin when co-incubated with four ciprofloxacin-resistant bacterial strains (Figure 1A–D). Specifically, treatment with ciprofloxacin at 30 times its MIC effectively reduced CFUs by approximately 3-Log_10_ in *E. coli* CX93T; however, this killing effect was completely abolished when pre-cultured with BZH for 3 h. Although BZH also weakened the bactericidal effect of ciprofloxacin against susceptible *E. coli* ATCC25922, it exhibited a diminished extent compared to resistant strain *E. coli* CX93T (Figure S2A). Therefore, we employed the ciprofloxacin-resistant strain *E. coli* CX93T as a model for subsequent investigations.

Given that ciprofloxacin targets DNA polymerase and exhibits a bactericidal effect, our investigation aimed to determine if BZH could also impact the survival rate of *E. coli* CX93T following exposure to various classes of antibiotics, including meropenem, kanamycin, ceftiofur, florfenicol, and tetracycline at 30 times its MIC. As a result, we observed that BZH effectively reduced the activity of three bactericidal antibiotics (Figure 1E–G), while showing no significant influence on the effectiveness of tetracycline and florfenicol. These two antibiotics are known for their bacteriostatic properties in selectively inhibiting protein synthesis (Figure S2B,C). In conclusion, our findings demonstrate that BZH renders bactericidal antibiotics ineffective against ciprofloxacin-resistant bacteria.

### 2.2. BZH Triggers High Level of Antibiotic Tolerance

Considering the possibility that bacteria’s reduced susceptibility to antibiotics may be attributed to resistance development, we conducted tests on *E. coli* CX93T’s MIC for ciprofloxacin, kanamycin, meropenem, and ceftiofur before and after co-incubation with BZH for 3 h (Table S2). The findings revealed that the MIC values for all drugs tested remained unchanged pre- and post-BZH induction, suggesting that BZH did not induce heritable antibiotic resistance. To further differentiate its effects, we assessed the impact of BZH on the minimum time needed for eliminating 99% of the bacterial population (MDK_99_). Our findings revealed a delay of 4 h in MDK_99_ timing following BZH incubation (Figure 1H). Considering that persistent bacteria maintained their original MDK_99_ compared to susceptible bacteria, it can be concluded that BZH triggered antibiotic tolerance instead of persistence.

### 2.3. Analysis of the Transcriptome in E. coli CX93T Following Exposure to BZH

**Figure 2:**
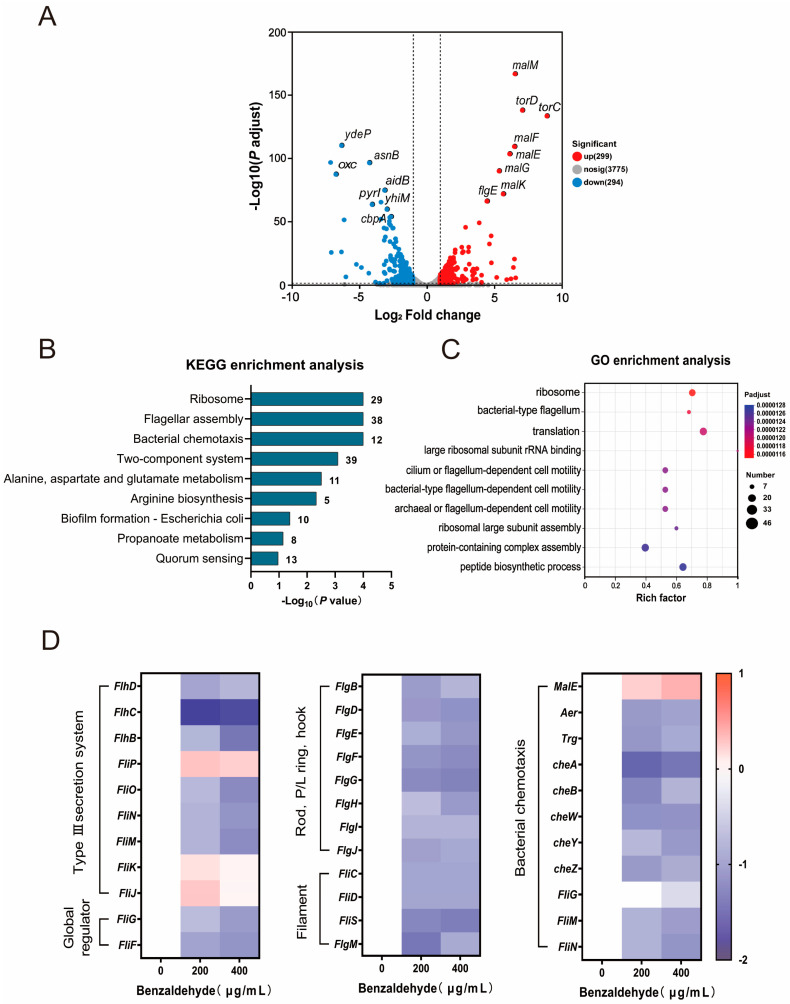
Transcriptome analysis of *E. coli* following exposure to benzaldehyde. (**A**) Volcano plot illustrating the differential expression of genes. The mRNA fold changes (Log_2_(FC)) in *E. coli* CX93T compared to *E. coli* CX93T treated with benzaldehyde, along with their corresponding significance values represented as log_10_ (*p* value). (**B**,**C**) Analysis of gene functional enrichment, including KEGG and GO enrichment; (**D**) RT-PCR results demonstrating the transcription levels of genes associated with bacterial flagella and chemotaxis.

To investigate the mechanisms underlying the increased tolerance to fluoroquinolones triggered by BZH, we conducted a transcriptome analysis of *E. coli* CX93T after exposure to BZH (400 μg/mL) for 3 h. Principal component analysis revealed significant distinctions between the groups treated with and without BZH. Notably, BZH treatment resulted in differential expression of genes, with 299 genes upregulated and 294 genes downregulated (fold change ≥ 2-fold, Figure 2A). The enrichment analysis based on Kyoto Encyclopedia of Genes and Genomes (KEGG) revealed that these differentially expressed genes were primarily associated with flagella assembly, bacterial chemotaxis, and two-component systems (Figure 2B). Similarly, the analysis of Gene Ontology (GO) enrichment revealed 276 biological processes associated primarily with the bacterial-type flagellum and flagellum-dependent motility (Figure 2C). As antibiotic tolerance is closely linked to metabolism, we utilized iPath3.0 for the visualization of significant metabolic pathways influenced by BZH treatment (accessed on 1 January 2024, https://pathways.embl.de/). The iPath analysis demonstrated alterations in numerous metabolic pathways upon exposure to BZH, with carbohydrate metabolism and energy metabolism particularly being the most prominent (Figure S3). To provide more direct evidence on the impact of BZH on bacterial flagella regulation, RT-PCR was performed to validate our transcriptome findings. Consistently with our results from RNA sequencing data analysis, functional genes related to bacterial flagella, including global regulator genes, type III secretion system genes as well as flagella protein-encoding genes such as *fliC*, *fliD*, *fliG*, *fliM*, *fliN*, etc., along with chemotactic-related genes *cheA*, *cheB*, *cheW*, *cheY*, and *cheZ*, were significantly downregulated upon BZH treatment (Figure 2D). In conclusion, our findings indicated that BZH-induced antibiotic tolerance is associated with changes in both flagellar regulation and metabolic processes within bacteria.

### 2.4. BZH Affects the Flagella Assembly and Motility

**Figure 3:**
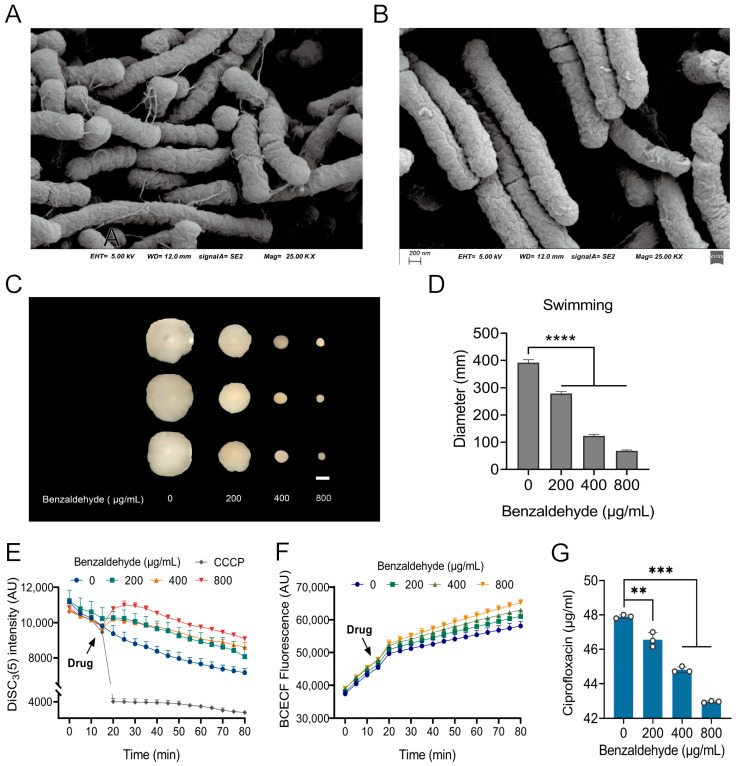
Benzaldehyde inhibits the formation of bacterial flagella and reduces intracellular antibiotic accumulation levels. (**A**,**B**) The surface morphology observed using scanning electron microscope in the absence (A) and presence (**B**) of benzaldehyde; (**C**,**D**) benzaldehyde inhibited swimming motility of *E. coli* CX93T in a dose dependent manner; (**E**,**F**) benzaldehyde dissipated the Δ*ψ* (**E**), while showed no effect on ΔpH; (**G**) benzaldehyde decreased the intracellular accumulation of ciprofloxacin. ** *p* < 0.01, *** *p* < 0.001, and **** *p* < 0.0001.

We initially employed scanning electron microscopy [24] to observe the impact of BZH treatment on flagella, using a concentration of 400 μg/mL (Figure 3A,B). Surprisingly, we found that the flagella were no longer visible after exposure to BZH. Since flagella play a crucial role in bacterial motility, we assessed the swimming ability of *E. coli* CX93T through a motility test. The observed diameter of the bacterial colonies decreased as the concentration of BZH increased (Figure 3C,D), indicating that BZH reduced bacterial motility. As bacterial motility is closely linked to proton motive force (PMF) integrity, we utilized BCECF-AM and DiSC_3_(5) fluorescent probes to observe the changes in two components of PMF: ΔpH and Δ*ψ*, correspondingly. Considering the positive control CCCP, which disrupted Δ*ψ* as evidenced by a decrease in fluorescence intensity, gradual elevation in fluorescence intensity was observed when cells probed with DiSC_3_(5) were exposed to BZH (Figure 3E), suggesting the disruption of Δ*ψ* through BZH treatment. However, introducing BZH into BCECF-AM-labeled cells did not significantly increase fluorescence levels (Figure 3F). Collectively, these results suggest that BZH interferes with the Δ*ψ* component of PMF. The PMF plays a vital role in drug intake and efflux processes, subsequently affecting intracellular drug accumulation levels. Therefore, we evaluated intracellular ciprofloxacin accumulation using an ELISA method and found that it decreased proportionally with increasing concentrations of BZH (Figure 3G). These results suggest that BZH inhibits the formation of bacterial flagella while releasing PMF for antibiotic efflux purposes, thereby reducing intracellular antibiotic accumulation levels.

### 2.5. BZH Decelerates TCA Cycle and Bacterial Metabolism

**Figure 4:**
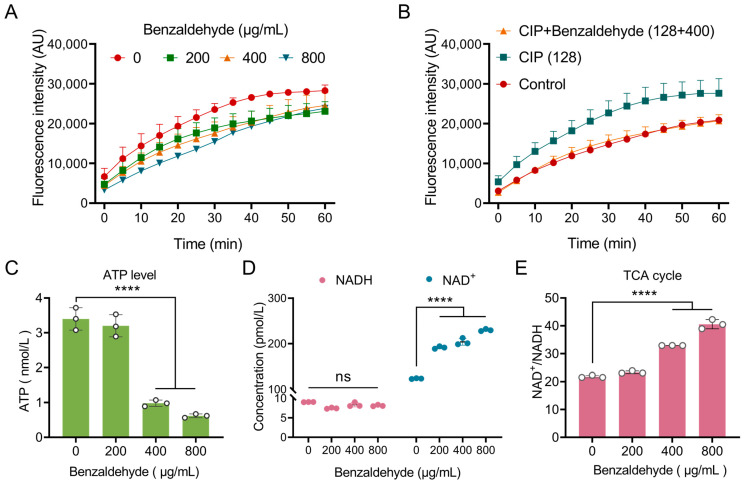
Benzaldehyde decelerates the TCA cycle and bacterial metabolism. (**A**) Benzaldehyde inhibited bacterial respiration; (**B**) the respiration rate increase induced through ciprofloxacin was inhibited by benzaldehyde; (**C**) benzaldehyde downregulated the intracellular ATP level. (**D**) Benzaldehyde increased the level of NAD^+^; (**E**) benzaldehyde increased the ratio of NAD^+^/NADH. The mean ± SD values presented in this study were derived from three separate experiments. Statistical analysis revealed no difference (ns *p* > 0.05) a highly significant difference (**** *p* < 0.0001).

According to the transcriptomics findings, a notable reduction was observed in the expression levels of genes associated with carbohydrate metabolism and energy metabolism. Consequently, resazurin was employed to monitor the respiration of *E. coli* CX93T. The addition of BZH for 60 min resulted in a suppression of the respiration rate (Figure 4A). Similarly, BZH inhibited the increased respiration rate caused by ciprofloxacin (Figure 4B). Furthermore, ATP production was significantly reduced by BZH (Figure 4C). In bacterial respiration, the TCA cycle involves dehydrogenation reactions and generates three NADH molecules as electron donors. Therefore, we evaluated the levels of NAD^+^/NADH. It was observed that BZH led to an accumulation of NAD^+^ (Figure 4D) and an elevated ratio of NAD^+^/NADH (Figure 4E), revealing a decelerated TCA cycle and bacterial metabolism due to BZH treatment.

**Figure 5:**
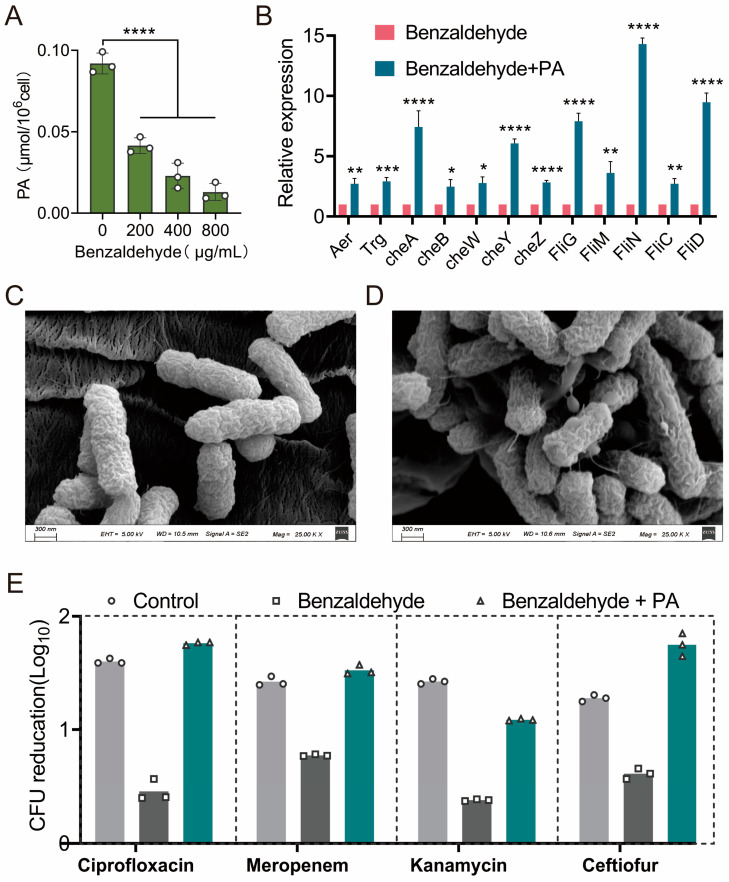
Pyruvate plays a critical role in benzaldehyde-induced tolerance. (**A**) The intracellular pyruvate concentration decreased in a manner dependent on the concentration of benzaldehyde; (**B**) the exogenous addition of pyruvate reversed the downregulation of genes related to bacterial flagella and chemotaxis caused by benzaldehyde; (**C**,**D**) the surface morphology of benzaldehyde-pretreated *E. coli* CX93T was examined using a scanning electron microscope in the presence (**D**) and absence (**C**) of pyruvate; (**E**) the exogenous addition of pyruvate successfully reinstated the bactericidal effect of antibiotics against benzaldehyde pretreated bacteria. The levels of significance were denoted as * *p* < 0.05, ** *p* < 0.01, *** *p* < 0.001, and **** *p* < 0.0001.

Pyruvate serves as a precursor to acetyl-CoA which initiates the TCA cycle. Consequently, the intercellular concentration of pyruvate underwent evaluation following exposure to BZH treatment. Notably, a decline in pyruvate concentration was observed in correlation with increasing concentrations of BZH (Figure 5A). Furthermore, the introduction of 10 mM pyruvate was found to effectively counteract the inhibitory effects caused by BZH on bacterial flagella assembly genes (*fliC*, *fliD*, *fliG*, *fliM*, and *fliN*) and chemotactic genes (*aer*, *trg*, *cheA*, *cheB*, *cheW*, *cheY*, and *cheZ*) expression (Figure 5B), and to restore flagellar formation (Figure 5C,D). The impact of bactericidal antibiotics on CFU was evaluated with the presence of exogenously added pyruvate. Encouragingly, the bactericidal effects of all the antibiotics were elevated (Figure 5E), indicating that the antibiotic tolerance triggered by BZH was reversed through the supplementation of pyruvate. In summary, BZH led to a reduction in intracellular pyruvate level and slowed down the TCA cycle and bacterial metabolism.

### 2.6. BZH Attenuates the Oxidative Damage of Bacteria Caused by Ciprofloxacin

**Figure 6:**
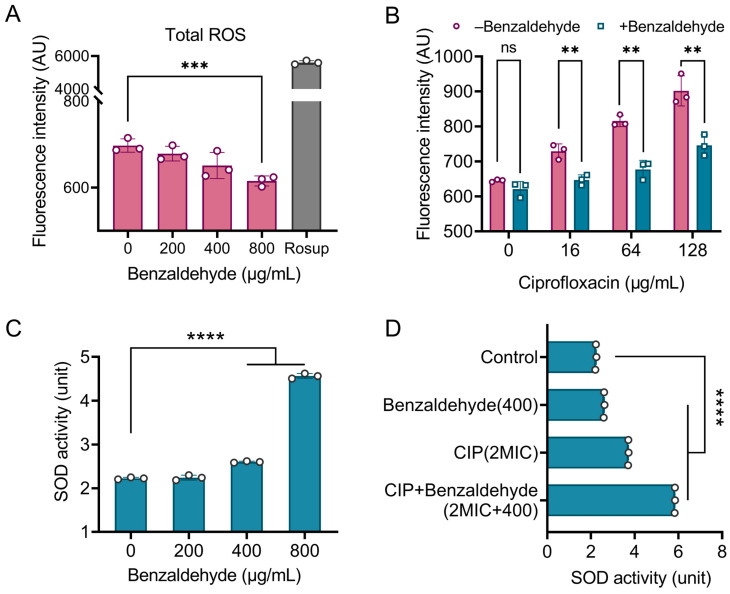
Benzaldehyde mitigates the oxidative damage caused by ciprofloxacin in bacteria. (**A**,**B**) The ROS level induced by benzaldehyde (**A**) or ciprofloxacin in combination with benzaldehyde (**B**); (**C**,**D**) the superoxide dismutase (SOD) activity under the treatment of benzaldehyde (**C**) or ciprofloxacin in combination with benzaldehyde (**D**). The levels of significance were denoted as ns *p* > 0.05, ** *p* < 0.01, *** *p* < 0.001, and **** *p* < 0.0001.

The formation of hydroxyl radicals is essential for the bactericidal effects of antibiotics. The decrease in metabolic rate results in a reduction in the production of reactive byproducts generated during metabolism, such as ROS. Growing evidence suggests that the formation of ROS plays a critical role in the bactericidal effects of antibiotics. Hence, we employed 2′,7′-dichlorofluorescein diacetate (DCFH-DA) to mark bacteria and examined the influence of BZH on the generation of ROS. Our results demonstrate that BZH effectively inhibits ROS production in *E. coli* CX93T (Figure 6A), thereby counteracting oxidative damage induced by ciprofloxacin exposure (Figure 6B). The activity of superoxide dismutase (SOD) was examined in the presence of BZH to assess its impact. The results demonstrated that the application of BZH led to a dose-dependent elevation in SOD activity (Figure 6C). Furthermore, this enhancement of SOD activity induced by BZH was also observed when ciprofloxacin was present (Figure 6D). These findings suggest that BZH disrupted the balance of the oxidation-antioxidant system and enhanced the bacteria’s antioxidant capacity, thereby reducing oxidative damage caused by ciprofloxacin.

### 2.7. BZH Diminishes the In Vivo Bactericidal Efficacy of Ciprofloxacin

**Figure 7:**
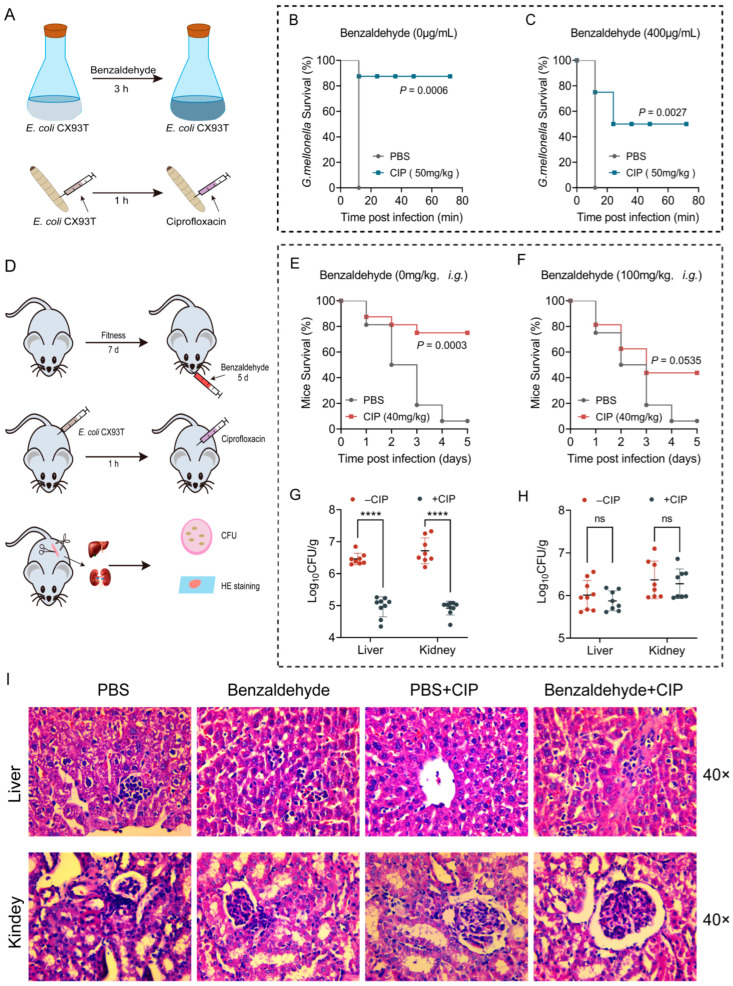
The in vivo effectiveness of ciprofloxacin is diminished upon administration of benzaldehyde. (**A**) Protocols for conducting experiments on the infection model of *G. mellonella* larvae. The survival rates of *G. mellonella* larvae (n = 12) infected with *E. coli* CX93T (10^5^ CFUs) were evaluated following co-culturing without (**B**) and with benzaldehyde (400 μg/mL, (**C**)) for a duration of three hours, followed by treatment with a single dose of ciprofloxacin (50 mg/kg). (**D**) Design of the experimental procedures for inducing peritonitis-sepsis infection in mice. (**E**,**F**) The survival rate curve of mice (n = 12) that were pre-administered with either PBS (**E**) or benzaldehyde (100 mg/kg, (**F**)) for a consecutive period of 5 days is depicted. Subsequently, the mice were intraperitoneally infected with *E. coli* CX93T and treated with either PBS or ciprofloxacin (40 mg/kg). (**G**,**H**) The levels of bacteria in the liver and kidney of mice, which were administered with PBS (**G**) or benzaldehyde (100 mg/kg, (**H**)) before infection as previously mentioned. (**I**) The HE staining results of liver and kidney under various treatment conditions. The levels of significance were denoted as ns *p* > 0.05 and **** *p* < 0.0001.

After showcasing the ability of BZH to enhance bacterial tolerance to antibiotics in laboratory settings, we proceeded to investigate whether BZH could also induce antibiotic tolerance in living organisms and consequently reduce the effectiveness of antibiotic treatment. To assess this, the *Galleria mellonella* infection models was used (Figure 7A). Insect larvae were subjected to infection with *E. coli* CX93T, which had been pre-cultured either in the presence or absence of BZH (400 μg/mL). Subsequently, a single dose of ciprofloxacin (50 mg/kg) was administered for treatment. As a consequence, the presence of BZH during co-cultivation decreased the bacteria sensitivity towards ciprofloxacin therapy, resulting in a notable decline in larval survival rate (Figure 7B,C). Additionally, we employed a mouse model of peritonitis-sepsis to evaluate the in vivo efficacy of ciprofloxacin (Figure 7D). Before being infected, mice were given either phosphate-buffered saline (PBS) or BZH (100 mg/kg) orally for five consecutive days. Afterwards, a lethal dose of *E. coli* CX93T (3.0 × 10^8^ CFUs) was administered intraperitoneally to all mice, followed by treatment with ciprofloxacin (40 mg/kg) or PBS. Consequently, the administration of a single dose of ciprofloxacin significantly enhanced the survival rate of mice over five days to 75% (*p* = 0.0003, Figure 7E) while also reducing bacterial loads in the liver and kidney compared to those observed in the control group (Figure 7F). On the contrary, the survival rate in the groups pre-administered with BZH decreased to 43% and the bacterial loads were nearly identical to those in the PBS control group (Figure 7H). Additionally, we conducted hematoxylin and eosin (HE) staining to assess any pathological alterations in the liver and kidney. Comparable pathological abnormalities, including the infiltration of inflammatory cells, were observed in both the BZH groups and PBS alone groups. However, such lesions were no longer present in the group treated with ciprofloxacin. Furthermore, it was noted that the BZH pretreated group exhibited similar inflammation changes as seen in the PBS group (Figure 7I), indicating that BZH may also play a role in enhancing antibiotics tolerance in murine infection models. Overall, it can be inferred from these findings that the therapeutic effectiveness of ciprofloxacin in animal infection models is impeded by BZH.

## 3. Discussion

With the advancement of molecular science, there has been a growing global concern over antibiotic tolerance, a phenomenon in bacteria where they remain phenotypically resistant but genetically susceptible to antibiotics. This greatly reduces the effectiveness of antibiotics in clinical settings [1,8,25]. What is alarming is that bacterial metabolites and external compounds have been found to induce antibiotic tolerance [18,26]. To enhance antibiotic efficacy and prevent antibiotic tolerance, it is crucial to investigate potential risks associated with everyday substances. Benzaldehyde (BZH) is commonly utilized as an antibacterial and antifungal preservative as well as a flavoring agent in food products, cosmetics, hygiene items, and pharmaceuticals [21]. In this study, we found that BZH exhibited a significant ability to induce high levels of antibiotic tolerance both in laboratory settings and in living organisms. Furthermore, the concentrations required for inducing antibiotic tolerance were lower than the approved limit for its use as a food additive. Similarly, two commonly used food additives, sodium dehydroacetate and potassium sorbate, have been documented to elicit bactericidal antibiotic tolerance [18,19]. In addition to food additives, previous studies have shown that the use of disinfectants such as phenol in daily life can induce widespread bacterial tolerance [27]. The consumption of a widely favored high-fat diet exhibits a positive correlation with the emergence of antibiotic tolerance [28]. These results emphasize the potential risks associated with the use of food additives and common household substances in relation to antibiotic tolerance.

The mechanistic investigations that we conducted have revealed that BZH-induced tolerance initially involves a downregulation in the expression of flagellar proteins, thereby inhibiting the formation of flagella. Previous research has demonstrated that *P. aeruginosa* biofilms with a knockout of the *flgE* gene, responsible for flagellar hook production, exhibit heightened tolerance to multiple antibiotics [17]. Similarly, another study found that *Salmonella* cells lacking flagella display increased antibiotic tolerance [16]. These findings indicated that a deficiency in flagellar-related proteins contributes to enhanced antibiotic tolerance. Further analysis indicated that BZH hampers bacterial motility and decreases the intracellular concentration of ciprofloxacin. It has been reported that flagellar motility relies on cellular energy stored as PMF and reduces efficiency in expelling toxic molecules like antibiotics [16]. This implies that BZH may impede both the assembly and rotation of flagella while consuming less PMF. The surplus PMF could enhance efflux activity and reduce intracellular antibiotic accumulation, thereby promoting antibiotic tolerance. This trade-off between motility and efflux underscores an innovative mechanism underlying antibiotic tolerance. However, the precise correlation between flagellar or motility attributes and antibiotic tolerance necessitates further investigations.

Additionally, BZH induces antibiotic tolerance by modulating the metabolic state of bacteria. The concentration-dependent decline in respiration and ATP levels, along with an elevated NAD^+^/NADH ratio, signify the inhibition of the TCA cycle. A prior investigation has documented that bactericidal antibiotics exert their effects by enhancing metabolic activity and causing collateral damage to intracellular macromolecules [5]. In contrast to bactericidal antibiotics, bacteriostatic agents induce largely opposing effects on bacterial metabolism [5,29]. This may explain why BZH inhibited the metabolism of bacteria cells and triggered tolerance for bactericidal antibiotics but not bacteriostatic agents. In a similar manner, phenazines enhance the tolerance of *P. aeruginosa* to ciprofloxacin by inducing increased metabolic heterogeneity within microaerobic biofilms [30]. Potassium sorbate, sodium dehydroacetate, and citric acid trigger tolerance by inhibiting bacterial metabolism [18,19,20]. The inhibition of the TCA cycle and activation of superoxide dismutase (SOD) activity induced by BZH impaired the reactive oxygen species (ROS) generation. It is widely acknowledged that antibiotic-induced cell death often occurs through oxidative damage mediated by ROS [26]. Therefore, BZH initiates tolerance by reducing intracellular ROS levels. Significantly, our findings indicate that exposure to BZH leads to a reduction in intracellular pyruvate levels. Interestingly, when supplemented with pyruvate, the tolerance induced by BZH was reversed. This aligns with the results reported by Yu-Bin Su et al., where the supplement of exogenous nitrogen sources like oxaloacetate, glutamate, and pyruvate to bacteria enhanced the effectiveness of aminoglycosides and provided respiratory energy [31]. These results emphasize the crucial role of the pyruvate cycle in antibiotic tolerance. Glucose, alanine, or lactate metabolism are the primary sources of pyruvate; whether their metabolisms also play a key role on BZH-induced tolerance needs further study. Thus, further investigation is required to gain a comprehensive understanding of how BZH remodels metabolism.

In conclusion, this study demonstrates that the widely utilized food additive BZH induces a high level of bactericidal antibiotics tolerance, especially fluoroquinolones, in resistant bacteria, both in vitro and in vivo. Firstly, BZH inhibits bacterial flagella formation and releases PMF to facilitate antibiotic efflux while reducing intracellular antibiotic accumulation. Secondly, BZH suppresses bacterial respiration and diminishes reactive oxygen species (ROS) generation. Thirdly, exogenous pyruvate effectively reverses BZH-induced tolerance and restores the efficacy of antibiotics, underscoring the pivotal involvement of the pyruvate cycle in antibiotic tolerance. Collectively, these findings underscore the critical involvement of BZH in protecting bacteria against antibiotic eradication and emphasize the urgent need for global attention regarding its widespread use as a food production additive. Furthermore, pyruvate holds promise as an adjuvant to enhance antibiotic effectiveness against tolerant strains.

## 4. Materials and Methods

### 4.1. Bacterial Strains and Chemical Reagents

The strains utilized in this investigation were documented in Table S1. Four multidrug resistant strains (*E. coli* CX93T *E. coli* PK8277, *E. coli* HH194M, and *E. coli* RB3-1) were isolated from birds, chickens, or a slaughterhouse, respectively, by our lab [32,33,34]. *E. coli* CX93T carry resistant genes such as *tet*(X4), *bla*_TEM-1_, *aac*, *aadA*, *aph*, *cat*, and *qnr*. Unless explicitly mentioned, bacterial strains were reactivated on Mueller–Hinton agar (MHA) and cultivated under aerobic conditions in MH broth (MHB) at a temperature of 37 °C with agitation at 200 rpm. Antibiotic medications were procured from the Institute of Veterinary Drug Control (Beijing, China). The remaining chemical substances were obtained from Aladdin (Shanghai, China) or Sigma-Aldrich (Oakville, ON, Canada).

### 4.2. Minimal Inhibitory Concentration (MIC) Determination

The minimum inhibitory concentrations (MICs) of BZH and antibiotics were assessed by employing a series of 2-fold dilutions of drugs, following the CLSI 2024 guideline [35]. The samples were incubated at a temperature of 37 °C for a duration of 18 h. The MIC values were determined as the lowest antibiotic concentrations that effectively inhibit bacterial growth.

### 4.3. Effect of BZH on Antibiotic Killing

Fresh MH broth (1 mL) was inoculated with overnight cultures of *E. coli* ATCC 25922, *E. coli* CX93T, *E. coli* PK8277, *E. coli* RB3-1, and *E. coli* HH194M at a dilution of 1/100 in the absence or presence of different concentrations of BZH (0–800 μg/mL). After a 3 h incubation period, the bacterial cells were gathered, rinsed thrice with PBS, and subsequently reconstituted in M9CA broth (Sangon Biotech, Shanghai, China). Subsequently, they were co-incubated with ciprofloxacin at a concentration equivalent to 30 times its MIC for an additional period of 5 h. Following this step, the aliquots of cell cultures were diluted in PBS and then spread onto MH agar plates. The bacterial load reduction was assessed by counting the colony-forming units (CFU) following a 12 h incubation at 37 °C subsequent to antibiotic treatment. To assess the impact of BZH on the bactericidal effect of different antibiotics against *E. coli* CX93T—including kanamycin, ceftiofur, and meropenem as bactericidal antibiotics, and tetracycline and florfenicol as bacteriostatic antibiotics—we followed similar procedures as described above with three independent biological replicates.

To explore the influence of pyruvate on BZH-induced antibiotic tolerance in *E. coli* CX93T, these cells were first pre-cultured with BZH (400 μg/mL) for a duration of three hours; subsequently, the cells were collected, rinsed, and re-suspended in M9CA broth before being exposed to ciprofloxacin at a concentration equal to thirty times its MIC along with pyruvate at a concentration level of 10 mM for another five-hour period. Finally, the remaining CFU counts were determined using the methods mentioned earlier to calculate corresponding reductions in bacterial count.

### 4.4. The Minimum Length of Time for 99% Killing Test

The duration needed to attain a 99% decline in the bacterial population (MDK99) was assessed through a time-based assay for bactericidal activity. In brief, the bacteria cultures were incubated overnight and subsequently diluted by a factor of 1/100 into fresh MHB, with or without the addition of BZH (400 μg/mL) for 3 h. Subsequently, the bacteria were washed thrice with PBS and suspended in M9CA medium, followed by the addition of ciprofloxacin at a concentration equivalent to 30 times its MIC. At different intervals (0, 2, 4, and 6 h), samples of the bacterial solution measuring 50 μL were collected for CFU determination. MDK_99_ was characterized as the minimum duration required to attain a 99% decrease in total CFUs from the initial inoculum at a particular concentration.

### 4.5. Transcriptomic Analysis

*E. coli* CX93T cultures were subjected to overnight incubation, followed by a 1/100 dilution into 10 mL of fresh MHB containing or lacking BZH (400 μg/mL), for a period of 3 h. Subsequently, the bacteria were gathered and rinsed three times with PBS. The R403 bacterial RNA extraction kit from Vazyme Biotech in Nanjing, China was utilized for the extraction of total RNA. The concentration and purity of the extracted RNA were assessed using NanoDrop 2000 spectrophotometry (Thermo Fisher Scientific, Waltham, MA USA). The integrity of the RNA was evaluated through agarose gel electrophoresis. The barcoded RNAs were subjected to reverse transcription to generate cDNA libraries, which were subsequently sequenced using the Illumina Hiseq 2000 system by Majorbio (Shanghai, China). After quality control, the raw data were subjected to a comparison with the reference genome in order to acquire mapped data (reads) for subsequent analysis. The functional annotation was conducted through a comparative analysis with the GO and KEGG databases. The gene expression levels were quantitatively analyzed using RSEM software v1.3.3, while DESeq2 was utilized to identify differentially expressed genes (DEGs).

### 4.6. RT-qPCR Analysis

*E. coli* CX93T was grown until it reached the stationary phase and subsequently diluted in a 1:1000 ratio into 2 mL of newly prepared MHB. The cultures were then incubated for a duration of 3 h, either with or without BZH (200, 400 μg/mL). After that, we utilized the Bacteria RNA Extraction Kit R403 (Vazyme, Nanjing, China) to isolate total RNA and assessed its concentration through the measurement of the absorbance ratio (260 nm/280 nm). The reverse transcription was performed using the PrimeScript RT Kit (Takara, Dalian, China) and gDNA Eraser (Takara, Dalian, China), in accordance with the manufacturer’s instructions. The ChamQ SYBR Color qPCR Master Mix (Vazyme, Nanjing, China) was utilized for RT-qPCR analysis, in conjunction with specific primers listed in Table S3, on a 7500 Fast Real-Time PCR System (Applied Biosystem, Foster City, CA, USA). The thermal cycling process involved a PCR amplification procedure consisting of 40 cycles at a temperature of 95 °C for 30 s, followed by denaturation at the same temperature for 5 s and annealing/extension at a temperature of 60 °C for 34 s. Using the 2^−ΔΔCt^ method with reference to a housekeeping gene (16S ribosomal RNA), we calculated the fold change in mRNA expression.

### 4.7. The Surface Morphology Observation with Scanning Electron Microscope [24]

*E. coli* CX93T (10^8^ CFU/mL) was exposed to either 0 or 400 μg/mL for 3 h. Then, the bacterial cells were gathered and rinsed with PBS for three times. The pellets were subsequently gently re-suspended in a pre-cooled glutaraldehyde solution at a concentration of 2.5% (*v*/*v*) for a duration of 24 h. The fixed strains underwent three additional washes with PBS, each lasting for approximately 10 min. Subsequently, an ethanol gradient method was employed to dehydrate the samples. To ensure complete drying, the samples were subjected to critical point drying and subsequently coated with spray-gold before being observed under a Gemini SEM 300 microscope(Carl Zeiss, Oberkochen, Germany).

### 4.8. Swimming Motility Assay

The swimming motility of bacteria was evaluated using the plate based assay [36]. A culture medium was prepared, which contained 0.3% agar and was supplemented with BZH at concentrations ranging from 0 to 800 µg/mL. A 2 μL bacterial suspension was carefully dispensed onto the center of a Petri dish and allowed to settle for a duration of 30 min. After being incubated at a temperature of 37 °C for a duration of 48 h, the size of the microbial colonies was measured.

### 4.9. The Intracellular Concentration of Ciprofloxacin

The ciprofloxacin rapid detection kit (ELISA, No. JPS-P100027), manufactured by Jepps in Shanghai, China, was employed to quantify the intracellular concentrations of ciprofloxacin. The limit of detection of this method was 0.1 ng/mL. *E. coli* CX93T was subjected to a 3 h culture, either with or without the addition of BZH (400 µg/mL). Then, the bacterial cells were gathered and re-suspended in PBS solution, achieving a concentration of 10^5^ CFU/mL. Ciprofloxacin was subsequently introduced at a concentration of 16 μg/mL and subjected to incubation for one hour at a temperature of 37 °C with agitation set at 200 rpm. The cells were collected and dissolved in 200 μL water. Subsequently, the bacterial cells underwent three freeze–thaw cycles by exposing them to liquid nitrogen and subsequently immersing them in a water bath maintained at a temperature of 65 °C. The collected supernatant was obtained by subjecting the mixture to centrifugation at a speed of 12,000 revolutions per minute for a duration of 10 min. Additionally, ciprofloxacin extraction from the pellet involved adding 500 µL acetonitrile. Finally, the combined supernatants were used for detecting ciprofloxacin concentrations as per the instructions provided with the ciprofloxacin rapid detection kit.

### 4.10. Bacterial Respiration Assay

*E. coli* CX93T was subjected to an overnight incubation at a temperature of 37 °C. Subsequently, the bacterial cultures were washed and resuspended in M9CA medium. The OD_600_ of the bacterial suspension was standardized to 0.5. This standardized suspension was utilized for measuring various biochemical parameters. Different concentrations of BZH (ranging from 0 to 800 µg/mL) or ciprofloxacin (at concentrations of 0, 16, 64, and 128 μg/mL) were individually added or used in combination (400 µg/mL BZH with varying concentrations of ciprofloxacin). Bacterial cells treated with BZH and ciprofloxacin alone or in combination was introduced to a well plate containing resazurin solution (Aladdin, Shanghai, China) at a concentration of 0.1 μg/mL. The fluorescence intensity was immediately assessed by employing an Infinite M200 Microplate reader (Tecan, Mannedorf, Switzerland) with an excitation wavelength of 550 nm and an emission wavelength of 590 nm over a period of 60 min.

### 4.11. Measurement of Intracellular ATP Levels

Subsequent to a 1 h pretreatment with ciprofloxacin or BZH, the *E. coli* CX93T culture was subjected to centrifugation, washing, and resuspension in lysis buffer. After lysing the sample, it was subjected to centrifugation at 12,000 rpm for 5 min at a temperature of 4 °C. The resulting supernatant was collected. The intracellular ATP levels was measured with the ATP Assay Kit (Beyotime, Shanghai, China) following the manufacturer’s meticulous instructions.

### 4.12. Measurement of NAD^+^ and NADH Levels

The NAD^+^/NADH Assay Kit (Beyotime, Shanghai, China) was employed to determine the levels of NAD^+^ and NADH in *E. coli* CX93T. Following pretreatment with BZH for 1 h, as previously described, the bacteria cells were collected and rinsed with PBS. Subsequently, the cell pellets were resuspended in 200 µL of a pre-chilled extraction buffer and subjected to centrifugation at 12,000 rpm for a duration of 10 min at a temperature of 4 °C. Finally, the supernatant was accurately quantified using NAD^+^/NADH Assay Kit incorporating WST-8 reagent (Beyotime, Shanghai, China).

### 4.13. The Measurement of PMF

The bacterial suspension of *E. coli* CX93T with the OD_600_ of 0.5 was labeled with BCECF-AM (5 µM) or 3,3′-dipropylthiadicarbocyanine iodide DiSC_3_(5) (0.5 µM) for the measurement of intracellular pH difference (ΔpH) and the electric potential (Δ*ψ*), respectively. Then, the labeled cells were treated with BZH for 1 h. The BCECF-AM-labeled cells were subjected to a monitoring of their fluorescence intensity using an excitation wavelength of 488 nm and emission wavelength of 535 nm. The Δ*ψ* was determined by recording the fluorescence intensity using an excitation wavelength of 622 nm and an emission wavelength of 670 nm with a time interval of 3 min for a duration of 80 min.

### 4.14. Total ROS Assay

The intracellular reactive oxygen species (ROS) level in *E. coli* CX93T was assessed using the 2′,7′-dichlorofluorescein diacetate (DCF-DA) dye. Following a pretreatment period of 1 h with BZH or ciprofloxacin plus BZH, as previously described, the ROS level was measured utilizing the ROS assay kit from Beyotime according to the manufacturer’s instructions. The measurement of fluorescence intensity was conducted using an excitation wavelength of 488 nm and emission wavelength of 525 nm.

### 4.15. SOD Activity Determination

To evaluate the intracellular superoxide dismutase (SOD) activity of *E. coli* CX93T after exposure to BZH or ciprofloxacin, we employed the Total Superoxide Dismutase Assay Kit with WST-8 (Beyotime, Shanghai, China) as per the manufacturer’s guidelines.

### 4.16. The Intracellular Concentration of Pyruvate

Intracellular pyruvate concentrations were determined using the pyruvate concentration detection kit (Jepps, Shanghai, China). *E. coli* CX93T was cultured at 37 °C, 200 rpm for 4 h with BZH ranging from 0 µg/mL to 800 µg/mL. Following this, bacterial cells were harvested and subjected to three washes with PBS. The resulting cell pellets were utilized for determining pyruvate concentrations as per the instructions provided with the detection kit.

### 4.17. The Animal Experiments

A total of 48 larvae of the species *Galleria mellonella* were acquired from Huiyude Biotech (Tianjin, China) and divided into four groups randomly. They were subsequently infected with a suspension of *E. coli* CX93T (10 μL, containing 1.0 × 10^5^ CFUs per larva), either alone or after being pre-cultured with BZH (400 µg/mL) for 3 h. After one hour of infection, the *Galleria mellonella* were administrated either PBS or ciprofloxacin at a dosage of 50 mg/kg. The *Galleria mellonella*’s survival rates were observed over a period of three days.

A group of 48 BALB/c male mice (6–8 weeks old) were obtained from the Comparative Medicine Centre at Yangzhou University in Jiangsu, China. The animals were allowed to acclimate for a period of one week prior to commencing the experiment. All animal procedures adhered strictly to the regulations and guidelines established by the Jiangsu Laboratory Animal Welfare and Ethical Committee of Jiangsu Administrative Committee of Laboratory Animals (ID: SYXK2022-0044). Subsequently, the mice received intraperitoneal injections of either PBS or BZH at a dosage of 100 mg/kg for five consecutive days. Following this treatment, they were inoculated with *E. coli* CX93T suspension containing approximately 3.0 × 10^8^ CFUs, intraperitoneally. After one hour of infection, the mice were randomly allocated into two groups: a control group and a ciprofloxacin treatment group, each comprising twelve mice. The control group was administered a single intraperitoneal dose of PBS, while the ciprofloxacin treatment group received ciprofloxacin at a dosage rate of 25 mg/kg. Survival rates were monitored for a duration of five consecutive days. The samples of liver and kidney were then collected under sterile conditions and separated into two portions for estimation purposes using CFU analysis or hematoxylin and eosin staining.

### 4.18. Statistical Analyses

GraphPad Prism Version 9.0 software was utilized for conducting the statistical analysis. A minimum of three biological replicates were employed to acquire all the data. The unpaired *t*-test and one-way analysis of variance (ANOVA) were used for comparing two groups and multiple groups, respectively, in in vitro investigations. The log-rank test and the Mann–Whitney U test were utilized to determine *p* values in in vivo studies. The levels of significance are indicated by asterisks as follows: ns, indicating no statistical significance; * *p* < 0.05, ** *p* < 0.01, *** *p* < 0.001, and **** *p* < 0.0001.

## Data Availability

All data generated or analyzed during this study are included in this published article (and its Supplementary Information Files).

## Supplementary Materials

The following supporting information can be downloaded at: https://www.mdpi.com/article/10.3390/ijms25168843/s1.
