# Supplementary material for: The Food Additive Benzaldehyde Confers a Broad Antibiotic Tolerance by Modulating Bacterial Metabolism and Inhibiting the Formation of Bacterial Flagella
Source: Int J Mol Sci. 2024 Aug 14;25(16):8843. doi: 10.3390/ijms25168843 (PMC11354442; doi:10.3390/ijms25168843)
Supplement: Supplementary file 1 [file ijms-25-08843-s001.zip › ijms-3129539-supplementary.pdf]

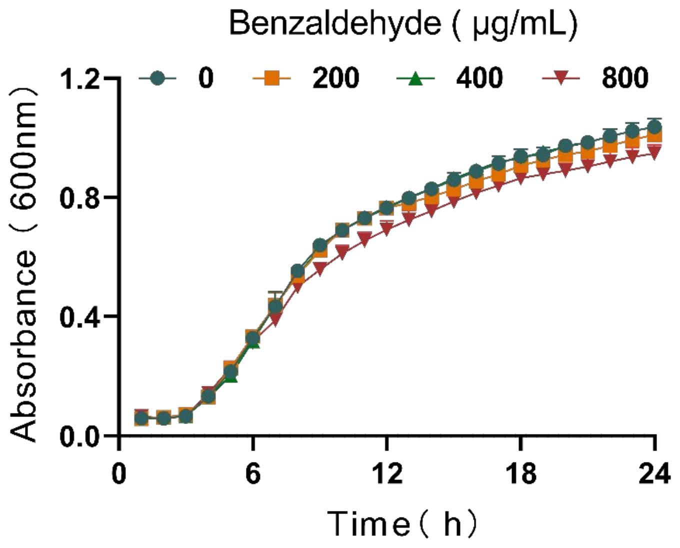

Figure S1 Bacterial growth curve of *E. coli* CX93T in the presence of Benzaldehyde

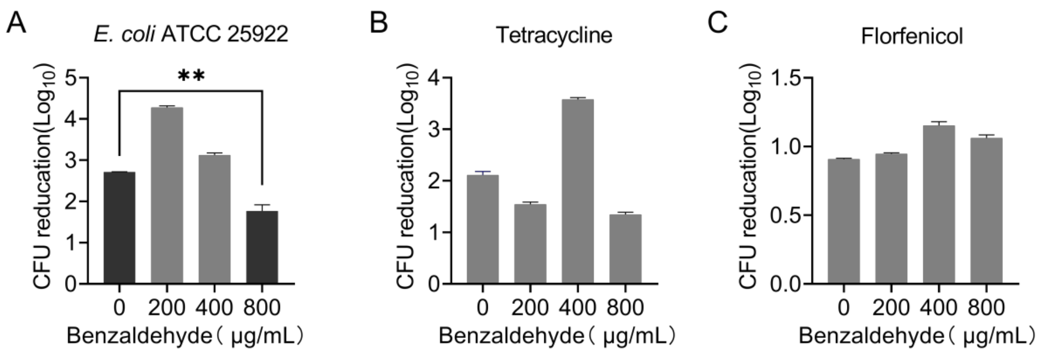

Figure S2 The influence of benzaldehyde on the bactericidal activity of antibiotics (A) Ciprofloxacin against *E. coli* ATCC25922; (B) Tetracycline against *E. coli* CX93T; and (C) Florfenicol against *E. coli* CX93T.

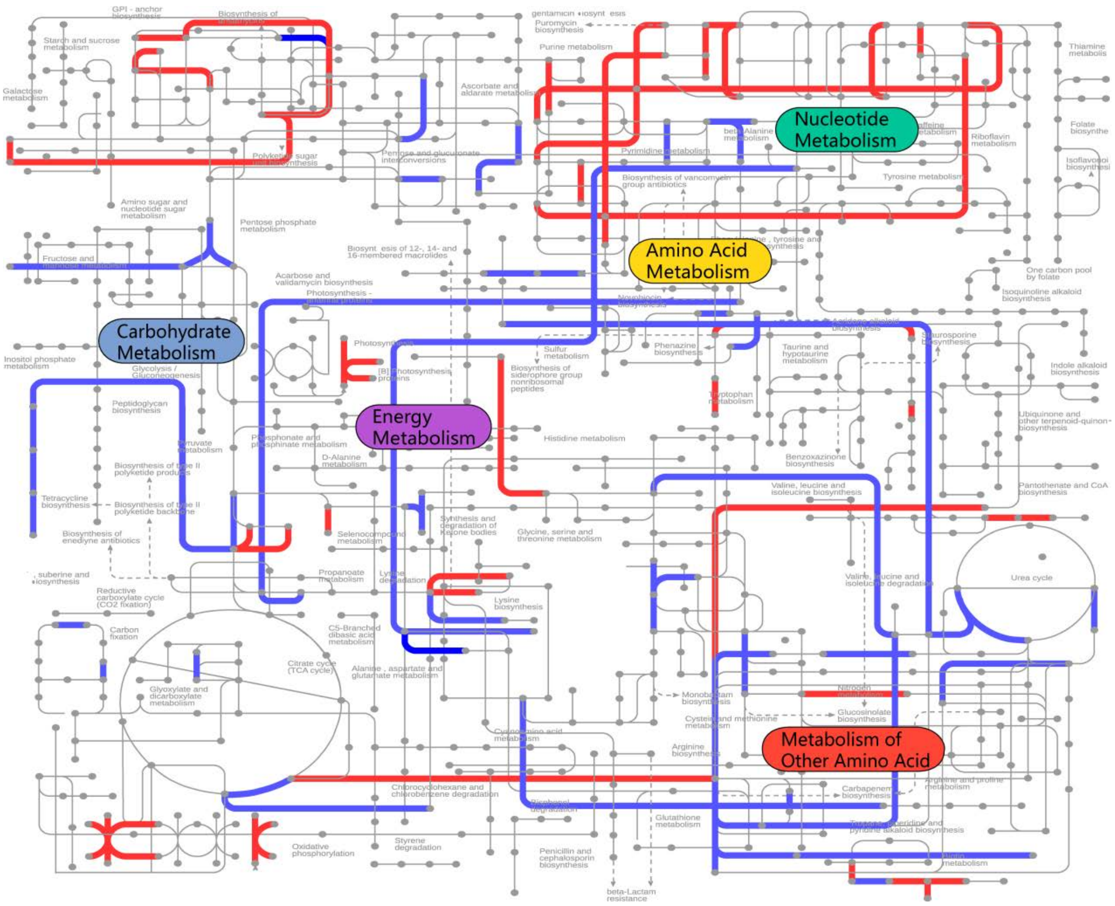

Figure S3 The Ipath analysis for global metabolic flux for *E. coli* CX93T under the treatment of Benzaldehyde

Table S1 The minimal inhibitory concentration of Ciprofloxacin and Benzaldehyde against pathogens used in this study

Pathogens	Genotype	MIC (μg/mL)	
		Ciprofloxacin	Benzaldehyde
E. coli CX93T	*tet*(B), *tet*(X4), *bla*$_{TEM-1}$, *bla*$_{EC-5}$, *aac*, *aadA*, *aph*, *cat*, *qnr*	16	>1024
E. coli PK8277	*mcr*-1, *tet*(A), *tet*(X4), *bla*$_{TEM-215}$	32	>1024
E. coli HH194M	*tet*(A), *bla*$_{TEM-1}$, *bla*$_{NDM-4}$, *bla*$_{EC}$	32	>1024
E. coli RB3-1	*tet*(A), *tet*(X4), *bla*$_{TEM-1}$, *bla*$_{CTX-M-14}$, *bla*$_{EC-8}$	16	>1024
E. coli ATCC 259 22	/	＜0.25	>1024
K.pneumoniae RGF105-1	*tmexCD3-toprJ1*	16	>1024

TableS2 MIC comparison of *E. coli* CX93T before and after Benzaldehyde induction

Antibiotics	MIC (μg/mL)[a]	MIC (μg/mL)[b]
Ciprofloxacin	16	16
Kanamycin	8	8
Meropenem	＜0.25	＜0.25
Ceftiofur	＞128	＞128

a, before benzaldehyde co-culture; b, after benzaldehyde co-culture.

Table S3 RT-qPCR primers used in this study

Genes	Sequence (5'→3')	Product (bp)
flgB	CGTATCTCGACGAACTGGGG	160
	CGTTTCGATAGGATCGGGCA	
flgD	CCCGAACACTGGCAAAGAGA	215
	GCCAGGGCGTCAGAAATGTA	
flgE	CCGCTTCCTACTGTTACG	237
	AGTGTTGTCGCTGTCTTC	
flgF	GGCAGCATTCAGGTTGAT	373
	CATCGGCAGCAATAGTGA	
flgG	GCACAGTCTTCCGAACAA	171
	ACCATCTGGCAACATCAC	
flgH	TATCAGCGGCAGCAATAC	129
	GGCGACAGGTTAAGGAAG	
flgI	TTCGGTGGTGATGAATCG	372
	TATCTGGCTGGCTGACAT	
flgJ	GCAATCACTCAACGAACTAA	185
	GCTGGTCATACATACTGGTA	
flgM	GCCTCTGAAGCCTGTAAG	197
	CGTTACGAATCGCCAGTT	
fliC	CGGTGGTGATAACGATGG	137
	CGCCTGAAGTGATAGTTGT	
fliD	AATGCCTACAACTCGCTAAT	304
	CTGGAAGAACTGACGGTATT	
fliS	ACATCATTGAGAACGGACTG	229
	TTCCAGGCATCGGCAATA	

Genes	Sequence (5'→3')	Product (bp)
fliF	AGACGAACAACTACAACAAC	458
	TATCCACTGGCGAATGAC	
fliG	ATCCTGCTGATGACCATTG	339
	AGATAATCGTTGGCGTTGA	
fliM	TATCTCGCTACGCCTGTC	272
	CGCATACTGACCGTTGAG	
fliN	GCGAACCACTGGATATTCT	285
	GCTCAGACGGAGTAATGATA	
fliO	GTTGTGGTGGTCGATGTG	250
	CGGTCTGCGGTATCTCTT	
flhB	GTGGTGCTTGGTGTCATT	434
	GAACTCATCACGAATATCCTG	
flhC	CCACAAGCAGAAGAAGGA	174
	GGATGGCGGTTGACATAA	
flhD	ACAGCCACCAGACGATTA	100
	TCATTCAGCAAGCGTGTT	
cheA	AGTGAACCGCAAGATGAG	349
	GAGTACCGCTGTGATGTC	
cheB	CAACAGCCATAGCGACAT	385
	TTCAACATCCAGCGTCAG	
cheW	TCAGGTAACACGGATTGC	152
	CGAGATTCAGGACGATAACT	
cheY	ATGTTGAGGAAGCGGAAG	199
	GCAGCAATGATGTTCTCTT	

Genes	Sequence (5’→3’)	Product (bp)
cheZ	TGGTAACAGATACACGACAA	137
	GACATCCATCATCCGCTTA	
aer	TGTGCGTTAGTGGTATTGT	216
	TGCGATTCAGATGCTCAA	
trg	ACTGCGGAGATTGAGATTC	439
	ATAACCTTGCTGCGACTG	
